# Rapid and Selective Determination of Folate Receptor *α* with Sensitive Resonance Rayleigh Scattering Signal

**DOI:** 10.1155/2017/1670812

**Published:** 2017-05-25

**Authors:** Liping Wu, Yue Liu, Rong Huang, Huawen Zhao, Weiqun Shu

**Affiliations:** ^1^Department of Environmental Hygiene, College of Preventive Medicine, Third Military Medical University, Chongqing 400038, China; ^2^Department of Chemistry, College of Pharmacy, Third Military Medical University, Chongqing 400038, China

## Abstract

A rapid, simple, and novel method for folate receptor *α* (FR*α*) determination is reported here. A probe of gold nanoparticles (Au NPs) modified with anti-FR*α* antibody was synthesized under the optimized conditions first. The antibody-modified Au NPs would aggregate when FR*α* was added to the probe for the specific interaction between antibody and antigen, resulting in the enhancement of resonance Rayleigh scattering (RRS) intensity. There is a linear relationship between the change of RRS intensity (Δ*I*_RRS_) and the concentration of FR*α*, with the detecting range of 0.50–37.50 ng·mL^−1^ and the limit of determination of 0.05 ng·mL^−1^. The determination of FR*α* in serum samples was realized with the advantages of high selectivity, high sensitivity, and easy operation.

## 1. Introduction

Folate receptors (FRs) are a family of glycoproteins on cell membrane [1]. Folate in tissues could be specifically recognized by folate receptors. There are three subtypes of folate receptors expressed on cells, which are FR*α* (also called FR1), FR*β*, and FR*γ*, respectively. Low level of folate receptors are expressed in normal cells or tissues. However, the expressing level of folate receptors, especially FR*α*, is greatly increased in most human tumors [2], to fulfill the need of massive folate for the proliferation of cancer cells [3, 4], indicating that the folate receptor could be served as a tumor biomarker for the initiation and progression of cancers and as a therapeutic target for cancer treatments [5]. The expressing level of folate receptor is extremely higher in colon cancers than in normal tissues [5]. Furthermore, the high expression of folate receptor is also associated with other tumors, such as lung cancer [6, 7], breast cancer [8], ovarian cancer [9–11], and brain tumor [5]. So the methodological basis could be provided for early diagnosis and monitoring of cancers by effective and quantitative determination of folate receptor.

Currently, methods for folate receptor determination have been reported, such as fluorescence quenching or imaging [12, 13], electrochemical or electrochemiluminescence biosensors [14–17], and colorimetric detection [18–20]. For these methods, fluorescent dyes, electrochemical luminescent dyes, or expensive instruments are needed, terminal protection of small-molecule-linked DNA should be done first, or the detection is not sensitive enough. So it is still significant to set up simple and rapid ways to determine FR*α* for the early monitoring of cancers.

In this contribution, FR*α* is determined using the distinctive resonance Rayleigh scattering (RRS) property of gold nanoparticles (Au NPs). RRS technology is well known for the high sensitivity and the convenience in performance and apparatus (common fluorophotometer). RRS method has been widely used to determine metal ions [21], biomolecules [22], medicines [23], pesticides [24], and so forth. Au NPs could be usually used as RRS probe for the special optical property. In our work, anti-FR*α* antibody was modified on the surface of Au NPs under optimized conditions, which was accomplished easily. In other words, Au NPs probe was made first. The antibody-modified Au NPs would aggregate when FR*α* was added to the probe, resulting in the enhancement of RRS intensity, for the specific interaction between anti-FR*α* antibody on the surface of Au NPs and FR*α* antigen. Meanwhile, there is a linear relationship between the change of RRS intensity (Δ*I*_RRS_) and the concentration of FR*α*, with the detecting range of 0.50–37.50 ng·mL^−1^, the limit of determination of 0.05 ng·mL^−1^, and the correlation coefficient of 0.9996. There are other methods reported for the determination of FR*α*. Compared to these methods, the advantages of our method are listed as follows. Firstly, the sensitivity of determination is guaranteed for RRS intensity is used as the response signal. Secondly, FR*α* could be determined with high selectivity, since it is based on the specific interaction between anti-FR*α* antibody and antigen in our method. Finally, the modification of anti-FR*α* antibody on the surface of Au NPs is simple to be carried out and the RRS performance is convenient to be done with a common and cheap fluorophotometer.

## 2. Experimental

### 2.1. Materials and Reagents

Au NPs were synthesized with Na_3_C_6_H_5_O_7_·2H_2_O and HAuCl_4_·4H_2_O. 1 mg·mL^−1^ Anti-FR*α* (anti-FOLR*α*, anti-FOLR1) polyclonal antibody was purchased from Sigma-Aldrich Corporation (Missouri, USA). 10 *μ*g·mL^−1^ of Anti-FR*α* working solution was diluted with sterile PBS buffer (0.01 M, pH 7.4). Lyophilized powder of folate receptor *α* (FOLR1, FR1, and FR*α*) was purchased from Sigma-Aldrich Corporation (Missouri, USA). 50 *μ*g·mL^−1^ of FR*α* stock solution was made by dissolving the lyophilized powder with sterile PBS buffer (0.01 M, pH 7.4). 1 *μ*g·mL^−1^ of working solution was diluted with the sterile PBS buffer. 10.0% BSA, 1.0% NaN_3_, 10.0% NaCl, and BR buffer are used when anti-FR*α* is modified on the surface of Au NPs.

### 2.2. Apparatus

RRS spectra and intensity were measured with a LS55 fluorescence spectrophotometer (Perkin Elmer, USA). Other instruments are the same as that in our previous work [25].

### 2.3. Synthesis of Anti-FR*α*-Antibody-Modified Au NPs Probe

The synthesis procedure of original Au NPs was mentioned in [25]. The concentration of Au NPs was calculated based on Lambert-Beer law; *A* = *εbc*. The extinction coefficient *ε* for 13-nm Au NPs is 2.7 × 10^8^ M^−1^·cm^−1^ [26]. The average size of Au NPs we synthesized is about 13 nm according to TEM images in Figure 2. So we use this extinction coefficient to calculate the concentration of Au NPs. The absorption intensity of Au NPs solution was 0.487 after fivefold dilution, which was detected in a 1-cm absorption cell. According to the equation *A* = *εbc*, the original concentration of Au NPs solution was calculated to be 9.0 nM. Afterward, Au NPs need to be modified with anti-FR*α* antibody, which can be served as a probe for FR*α* determination. The principle and the detailed method of how we optimize the condition of pH and antibody concentration were described in [25], but the concentrations of certain substances are different. For pH optimization in this contribution, 200 *μ*L of Au NPs solution with onefold dilution, 35 *μ*L of doubly distilled water, 30 *μ*L of BR buffer with certain pH, and 15 *μ*L of 10 *μ*g·mL^−1^ anti-FR*α* antibody were added. Then 20 *μ*L of 10.0% NaCl was added to test the stability of the antibody-modified Au NPs on certain pH conditions. The optimizing process of anti-FR*α* antibody was carried out in the same way.

Anti-FR*α*-modified Au NPs probe was synthesized according to the optimal conditions we got above. Basically, 20.0 mL of Au NPs solution with onefold dilution, 3.0 mL of BR buffer (pH 7.00), and 1.2 mL of 10 *μ*g·mL^−1^ anti-FR*α* antibody were adopted when Au NPs were modified with anti-FR*α*. The probe was washed twice and resuspended in 20.0 mL of sterile PBS buffer with slight BSA and NaN_3_, finally.

### 2.4. Procedure in Detail for FR*α* Determination

First, the interaction between antibody-modified Au NPs probe and FR*α* was carried out in a 1.5 mL EP tube. 50 *μ*L of well-modified Au NPs probe solution and various concentrations of FR*α* working solution were added to EP tubes. Second, different volume of doubly distilled water was added to keep the total volume of 400 *μ*L, with mixing thoroughly and keeping the tubes at room temperature for 10 min. The color of the mixture changed from light red to light blue. Third, RRS spectra and intensity were measured with the detecting wavelength range of 500 nm to 700 nm, for there is a characteristic RRS peak within this range. RRS signal was obtained using a LS55 fluorescence spectrophotometer, by means of synchronous scanning at Δ*λ* = 0 (*λ*_ex_ = *λ*_em_) with slit width of 10 nm.

### 2.5. Pretreatment and Determination for Real Samples

The serum sample was obtained from the Southwest Hospital in Chongqing and stored at 4°C. The serum was diluted 10 times with sterile PBS (0.01 M, pH 7.4) before use. Two different concentrations of standard FR*α* were added to the serum samples, respectively. And then the determination was realized by calculating the recovery of standard addition.

## 3. Results and Discussion

### 3.1. Characteristics of RRS Spectra and TEM Images

**Figure 1:**
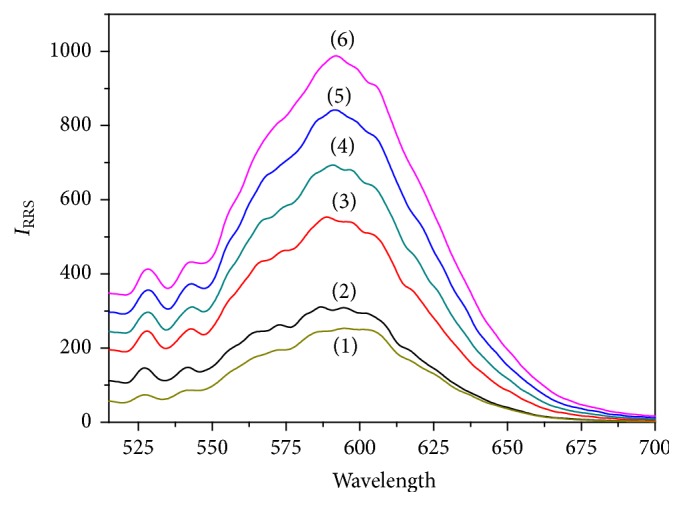
RRS spectra for the interaction between antibody-modified Au NPs probe and FR*α*. Curve 1, original Au NPs; Curve 2, antibody-modified Au NPs probe; Curves 3–6, interaction between the probe and FR*α*. Concentrations: FR*α* (Curves 3–6, ng·mL^−1^), 2.50, 12.50, 25.00, and 37.50.

RRS spectra of the interaction between antibody-modified Au NPs and FR*α* are shown in Figure 1. Curves 1 and 2 represent the original Au NPs and antibody-modified Au NPs, respectively. What we can see from Curves 1 and 2 is that RRS spectra of the original Au NPs and antibody-modified Au NPs are slightly distinct, which is because the surface of Au NPs had been changed after Au NPs were modified with anti-FR*α* antibody. A characteristic RRS peak is located at 590 nm when the interaction occurs between FR*α* and antibody-modified Au NPs probe (from Curve 3 to Curve 6). And the characteristic RRS intensity is enhanced gradually with the increasing concentration of FR*α*. So the quantitative determination of FR*α* is set up, based on the linear relationship between the change of RRS intensity and FR*α* concentration.

**Figure 2:**
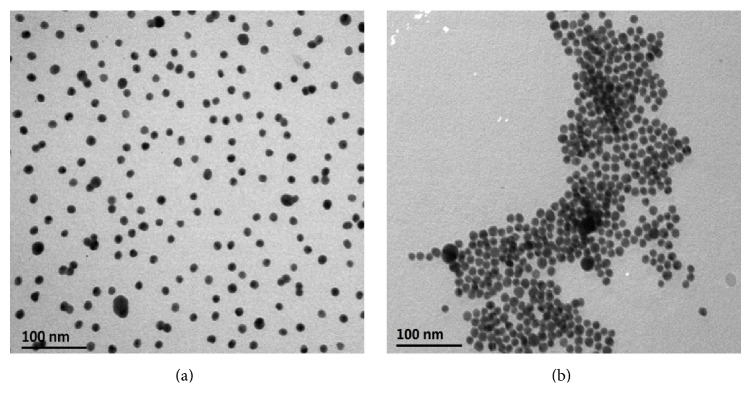
TEM images for antibody-modified Au NPs probe (a) and the interaction between the probe and FR*α* ((b) concentration of FR*α*, 12.50 ng·mL^−1^).

The distance among antibody-modified Au NPs was shortened and antibody-modified Au NPs aggregated when FR*α* was added, for the specific interaction between antibody and antigen. The more FR*α* was added, the stronger RRS intensity would be, within the certain concentration range of FR*α*. The enhancement of RRS intensity when FR*α* is added to the antibody-modified Au NPs probe is essentially caused by the aggregation of Au NPs, which could be proved by TEM images (Figure 2). It is shown that the antibody-modified Au NPs are dispersed well (Figure 2(a)) but aggregate dramatically when FR*α* is added (Figure 2(b)).

### 3.2. Optimization of pH and Antibody Concentration When Au NPs Were Modified with Anti-FR*α* Antibody

**Figure 3:**
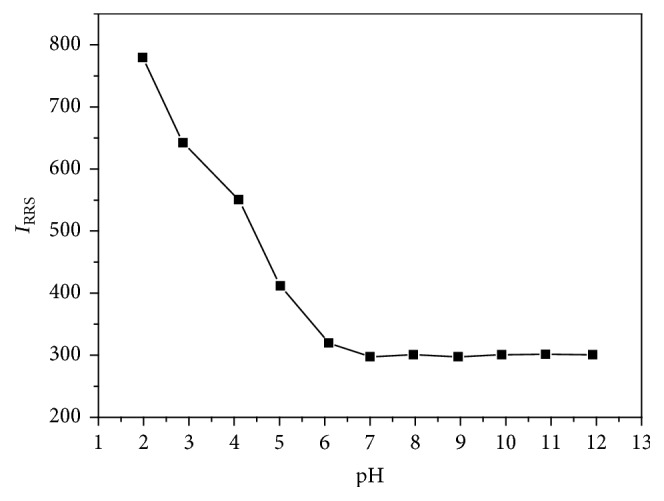
Effect of pH condition on the modification of Au NPs with anti-FR*α* antibody. Concentrations: Au NPs, 3.0 nM; anti-FR*α*, 0.50 *μ*g·mL^−1^; NaCl, 0.67%; pH, 1.98, 2.87, 4.10, 5.02, 6.09, 7.00, 7.96, 8.95, 9.91, 10.88, and 11.92.

The process of Au NPs modification with anti-FR*α* antibody could be affected by pH of the buffer. So pH condition was optimized first. The well-modified Au NPs would not aggregate and RRS intensity would not increase with relatively high concentration of NaCl and the appropriate pH condition was got under this principle. Anti-FR*α* antibody was added to Au NPs solutions at different pH conditions. After mixing and incubating, NaCl solution was added to the mixture. It is shown in Figure 3 that Au NPs aggregate and RRS intensity is increased with the addition of NaCl when pH is less than 6.09 and RRS intensity remains stable when pH is higher than that value, which demonstrates that Au NPs could be well modified under neutral and alkaline conditions. Finally, a neutral pH of 7.00 is chosen based on the result.

**Figure 4:**
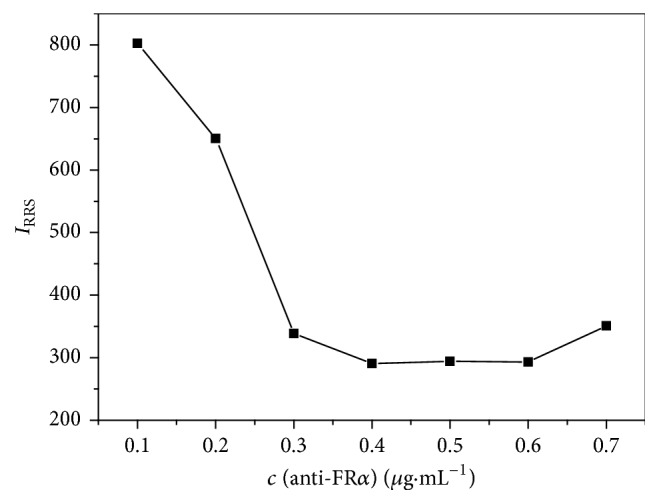
Effect of antibody concentration on the modification of Au NPs with anti-FR*α* antibody. Concentrations: Au NPs, 3.0 nM; pH, 7.00; NaCl, 0.67%; anti-FR*α* (*μ*g·mL^−1^), 0.10, 0.20, 0.30, 0.40, 0.50, 0.60, and 0.70.

The concentration of antibody must be taken into account as well when Au NPs were modified with anti-FR*α* antibody. The modification efficiency would be affected by the antibody concentration, which was described in this part. It is shown in Figure 4 that RRS intensity is increased with the addition of NaCl when the antibody concentration is less than 0.40 *μ*g·mL^−1^, which is because the whole surface of Au NPs could not be adsorbed thoroughly with fewer antibodies. However, RRS intensity stays stable when the antibody concentration is over the range of 0.40–0.60 *μ*g·mL^−1^, indicating that Au NPs could be well modified under this condition. Considering the losses in the real process of operation, we choose 0.50 *μ*g·mL^−1^ as the optimal concentration of anti-FR*α* antibody.

### 3.3. Selectivity for FR*α* Determination

We analyze the impact of the coexisting substances listed in Table 1 to investigate the selectivity for the determination of FR*α*. FR*α* with the final concentration of 25.00 ng·mL^−1^ and a certain concentration of a coexisting substance were added to the determination system. The change in RRS intensity was compared with the situation without the coexisting substance. What is shown in Table 1 is that the foreign substances, such as saccharides, proteins, amino acids, and metal ions, would not affect the detection of FR*α*, for the change in RRS intensity at 590 nm is within the permissible range. Theoretically, the determination for FR*α* is realized through the interaction between antibody and antigen. Anti-FR*α* antibody modified on the surface of Au NPs could specifically recognize FR*α*, which guarantees the high selectivity of FR*α* determination.

### 3.4. Linear Relationship and the Determination for Real Samples

**Figure 5:**
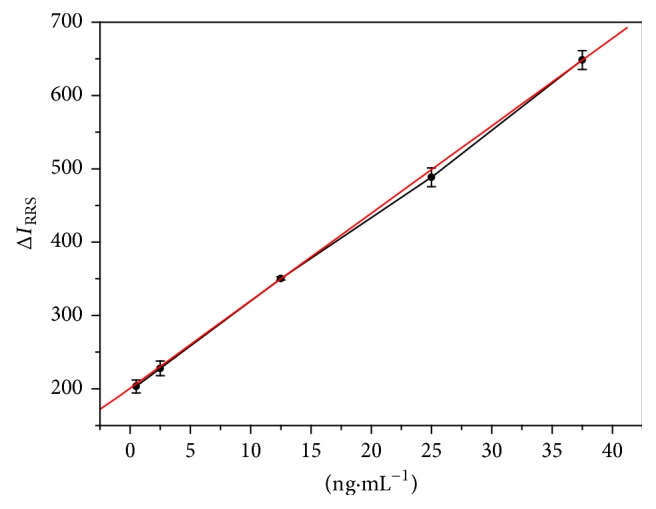
Calibration curve for FR*α* determination with error bars for SD from three independent measurements. Concentrations: FR*α* (ng·mL^−1^), 0.50, 2.50, 12.50, 25.00, and 37.50.

The standard calibration curve is illustrated in Figure 5, showing the result of the enhancement of RRS intensity Δ*I*_RRS_ against the FR*α* concentration. The quantitative determination of FR*α* is set up according to the linear relationship between Δ*I*_RRS_ and FR*α* concentration over the range of 0.50–37.50 ng·mL^−1^, with the linear regression equation, Δ*I*_RRS_ = 200.72 + 11.93*c*. The limit of determination (LOD) is 0.05 ng·mL^−1^, and the correlation coefficient is 0.9996.

To further validate the accuracy and feasibility of the method presented here, we determined the concentration of FR*α* in two real samples with ten parallel repeats for each one. The determination was realized by calculating the recovery of standard addition in diluted human serums, which is shown in Table 2. The detection recovery is between 90.88 and 105.59% and RSD is 4.12% and 2.91%, respectively. What we can see from the result is that the method is accurate and applicable to quantitatively determine FR*α* in serums.

## 4. Conclusion

In this contribution, anti-FR*α* antibody was stably modified on the surface of Au NPs under optimal conditions, so that effective Au NPs probe was synthesized for the determination of the tumor biomarker FR*α*. The quantitative determination of FR*α* is realized with resonance Rayleigh scattering signals, according to the linear relationship between Δ*I*_RRS_ and FR*α* concentration, with the advantages of easy operation, high sensitivity, and excellent selectivity.

## Figures and Tables

**Table 1:** Effect of coexisting substances on the FR*α* determination.

Coexisting substances	Conc.	Change in RRS intensity (%)	Coexisting substances	Conc.	Change in RRS intensity (%)
	(*μ*g·mL^−1^)			(*μ*g·mL^−1^)	
Sucrose	85.58	+1.02	Vc	0.25	−2.63
Glucose	45.04	+0.93	Histone	2.50	+3.21
Lactose	85.58	+0.85	Myoglobin	5.00	+1.95
Starch	25.00	+1.08	K^+^	4.88	−2.62
His	0.39	+3.18	Cu^2+^	0.79	−1.14
Pro	2.87	−1.39	Al^3+^	0.67	+2.99
Thr	2.98	+2.19	Zn^2+^	1.63	+1.08
Phe	4.13	+0.98	Mg^2+^	0.60	+1.52
Cys	3.03	+1.85	NH_4_^+^	45.00	+4.23
BSA	12.50	+1.28			

**Table 2:** Determination of FR*α* in real samples (*n* = 10).

Sample number	FR*α* added (ng·mL^−1^)	FR*α* detected (ng·mL^−1^)	Recovery (%)	RSD (%)
1	12.50	12.69	93.77–105.59	4.12
2	25.00	23.64	90.88–98.64	2.91
